# Diagnose- und Aufnahmezentrum: Etablierung und Evaluation einer integrierten translationalen Infrastruktur für die klinisch-psychiatrische Forschung

**DOI:** 10.1007/s00115-024-01609-5

**Published:** 2024-02-21

**Authors:** Urs Braun, Oliver Hennig, Johanna Forstner, Sarah Gerhardt, Mirjam Deffaa, DAZ-Team (die Mitglieder sind am Beitragsende aufgelistet), Dusan Hirjak, Michael Deuschle, Anne Koopmann, Christian Wisch, Melanie Fritz, Gabriele Ende, Heike Tost, Peter Schöfer, Stefan Bischoff, Matthias Janta, Falk Kiefer, Christian Schmahl, Tobias Banaschewski, Andreas Meyer-Lindenberg

**Affiliations:** 1 Zentralinstitut für Seelische Gesundheit, Medizinische Fakultät Mannheim, Universität Heidelberg, J5, 68159 Mannheim, Deutschland; 2 Deutsches Zentrum für Psychische Gesundheit (DZPG), Mannheim-Heidelberg-Ulm, Deutschland

**Keywords:** Diagnose- und Aufnahmezentrum, Klinisch-wissenschaftliche Phänotypisierung, Behandlungsoptimierung, RDoC, Personalisierte Psychiatrie, Diagnosis- and Admission Center, Broad phenotypting, Treatment optimiziation, RDoC, Personalized psychiatry

## Abstract

Die routinemäßige, tiefgreifende Charakterisierung von Patienten mit Methoden der klinischen und skalenbasierten Untersuchung, der Neuropsychologie, anhand von Biomaterialien und sensorbasierten Informationen verspricht transformative Möglichkeiten auf dem Weg zu einer personalisierten Diagnostik, Therapie und Prävention in der Psychiatrie, Psychotherapie und Psychosomatik. Die effektive Integration des zusätzlichen zeitlichen und logistischen Aufwands in den Versorgungsalltag sowie die Akzeptanz bei Patienten sind entscheidend für den Erfolg eines solchen Ansatzes, hierzu liegen jedoch bisher kaum Daten vor. Wir berichten hier über die Etablierung eines Diagnose- und Aufnahmezentrums (DAZ) am Zentralinstitut für Seelische Gesundheit (ZI) in Mannheim. Beim DAZ handelt es sich um eine den anderen Versorgungstrukturen vorgeschaltete ambulante Einheit zur klinischen und wissenschaftlichen diagnoseübergreifenden Phänotypisierung als Ausgangsbasis für eine datenunterstützte, individuelle Bahnung der weiteren Behandlungs‑, Diagnostik- oder Studienpfade. Wir beschreiben die Funktionen, Ziele und Implementierung der neu geschaffenen klinisch-wissenschaftlich translationalen Struktur, geben einen Überblick über die damit erreichten Patientenpopulationen und liefern Daten zur Akzeptanz. Die enge Verzahnung mit den nachgelagerten klinischen Prozessen ermöglicht dabei eine besser abgestimmte und bedarfsorientierte Zuweisung und einen schnelleren Beginn der störungsspezifischen Diagnostik und Therapie. Seit dem Start im April 2021 bis Ende 2022 wurden in einer Pilotphase 1021 Patienten im DAZ psychiatrisch untersucht. Die Patientenklientel entsprach dabei einer repräsentativen Stichprobe aus der Regelversorgung und die neu etablierten Prozesse wurden von Patienten als hilfreich erlebt. Zusammenfassend verknüpft das DAZ somit in hohem Maße Interessen und Bedürfnisse der Patienten mit der Erhebung wissenschaftlich relevanter Daten.

## Abstract (en)

The routine in-depth characterization of patients with methods of clinical and scale-based examination, neuropsychology, based on biomaterials, and sensor-based information opens up transformative possibilities on the way to personalized diagnostics, treatment and prevention in psychiatry, psychotherapy, and psychosomatics. Effective integration of the additional temporal and logistical effort into everyday care as well as the acceptance by patients are critical to the success of such an approach but there is little evidence on this to date. We report here on the establishment of the Diagnosis and Admission Center (DAZ) at the Central Institute of Mental Health (ZI) in Mannheim. The DAZ is an outpatient unit upstream of other care structures for clinical and scientific phenotyping across diagnoses as a starting point for data-driven, individualized pathways to further treatment, diagnostics or research. We describe the functions, goals, and implementation of the newly created clinical scientific translational structure, provide an overview of the patient populations it has reached, and provide data on its acceptance. In this context, the close integration with downstream clinical processes enables a better coordinated and demand-oriented allocation. In addition, DAZ enables a faster start of disorder-specific diagnostics and treatment. Since its launch in April 2021 up to the end of 2022, 1021 patients underwent psychiatric evaluation at DAZ during a pilot phase. The patient sample corresponded to a representative sample from standard care and the newly established processes were regarded as helpful by patients. In summary, the DAZ uniquely combines the interests and needs of patient with the collection of scientifically relevant data.

## Hintergrund und Fragestellung

### Klinischer Nutzen

Unser verbessertes Verständnis von psychischer Gesundheit und Erkrankungen führt zu einer zunehmenden Ausdifferenzierung von Behandlungsangeboten. Dies ermöglicht es, spezifischer auf einzelne Störungsbilder und häufige Kombinationen dieser einzugehen, im Sinne einer zunehmend individualisierten Therapie. Die durch wissenschaftliche Ausdifferenzierung und Wettbewerb entstandenen unterschiedlichen Schwerpunkte verschiedener psychiatrisch-psychotherapeutischer Einrichtungen erhöhen die Komplexität einer individualisierten Diagnostik- und Therapieentscheidung [8].

In der realen Versorgung stellt dies alle Beteiligten vor die Herausforderung, aus dem breiten Spektrum störungsspezifisch optimierter Therapien einen möglichst ideal auf die reale Person, ihre Erkrankung, Ressourcen und Bedürfnisse zugeschnittenen Behandlungsplan zu erstellen, nicht zuletzt unter Einbezug der Dringlichkeit sowie der Verfügbarkeit der Angebote.

Digitalisierung im Gesundheitswesen mit ihrem impliziten Anreiz der Zentralisierung und strukturellen Abstimmung von Prozessen bietet dabei die Chance, Reorganisation und Informationstechnologie zu nutzen, um Inhalte und Kapazitäten von Angeboten bedarfsorientiert zu definieren, anzupassen und neben optimierter Behandlung auch eine optimierte Auslastung und damit Ressourcennutzung zu gewährleisten.

### Wissenschaftlicher Nutzen

Wissenschaftlich stellt die datengestützte Prognose von Diagnostik, Behandlung und Verlauf psychischer Erkrankungen durch die Möglichkeiten maschineller Lernverfahren und künstlicher Intelligenz zunehmend eine Kernfrage klinisch-psychiatrischer Forschung dar [4]. Dabei gewinnt die Sekundärnutzung von Routinedaten sowie die zunehmende transdiagnostische Phänotypisierung von Patienten zunehmend an Bedeutung [7]. Der Erfolg dieser Analysemethoden ist maßgeblich abhängig von der Verfügbarkeit und Qualität großer und vereinheitlichter Datenmengen in Bio- und Medizindatenbanken, welche häufig nur im Rahmen großer Kollaborationen vieler Standorte möglich sind. Dieser Vernetzungsaspekt birgt dabei besondere Anforderungen an die technische Umsetzung einer sicheren und kompatiblen gegenseitigen Datennutzung durch sog. Datenintegrationszentren sowie die klinische Erfassung der Daten und der damit verbundenen Einholung des Einverständnisses der Patienten zur wissenschaftlichen Sekundärnutzung ihrer klinischen Routinedaten. Diese Bemühungen werden vom Gesetzgeber durch neue Fördermaßnahmen unterstützt, insbesondere durch die Medizininformatikinitiative (MII; [5]). Die MII hat unter anderem maßgeblich die rechtlichen und ethischen Rahmenbedingungen für eine Einverständniserklärung für eine breitere Forschungsdatennutzung im Sinne eines Broad Consent (BC) geschaffen. Dieser modular aufgebaute MII Broad Consent erlaubt, nach Votum durch die lokale Ethikkommission a) die Sekundärnutzung aller klinischen Routinedaten, b) die Verknüpfung mit Krankenkassendaten, c) die Rekontaktierung und d) die Nutzung und – unter spezifischen Voraussausetzungen – zusätzliche Gewinnung einer geringen (Zusatz‑)Menge an Biomaterialien [12].

Aufbauend auf dieser Infrastruktur ist einer der Schwerpunkte des Deutschen Zentrums für Psychische Gesundheit (DZPG) die inhaltliche und strukturelle Harmonisierung der für die psychiatrische Klinik und Forschung relevanten Datenmodalitäten über die Standorte hinweg [10]. Dies stellt insbesondere für sprechende Fächer wie die Psychiatrie und Psychosomatik mit ihrem starken Schwerpunkt auf ein multiprofessionales Therapeutenteam eine besondere Herausforderung dar.

## Studiendesign und Untersuchungsmethoden

### Diagnose- und Aufnahmezentrum (DAZ) am ZI Mannheim

**Figure 1:**
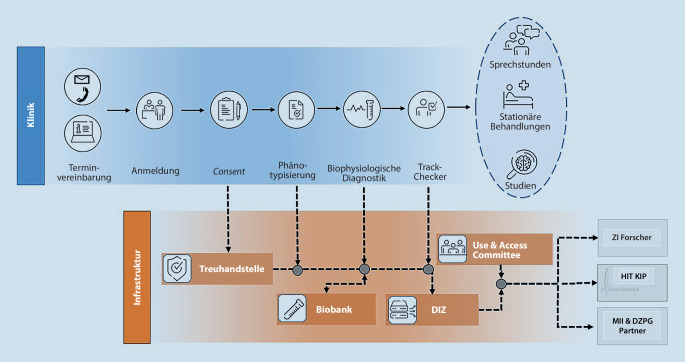

Um diesen Herausforderungen zu begegnen, wurde am Zentralinstitut für Seelische Gesundheit (ZI) in Mannheim mit dem Aufbau einer zentralisierten, klinisch integrierten und translationalen (im Sinne einer engen wechselseitigen Interaktion von Forschung und Klinik) Struktur zur Verbesserung der Versorgung und Forschung begonnen. Kerngedanke dieser den anderen Versorgungsstrukturen vorgeschalteten ambulanten Einheit ist es, eine gemeinsame sektoren- und klinikübergreifende Anlaufstelle für alle Patienten zu schaffen, die nach einer diagnoseübergreifenden Diagnostik einen Orientierungspunkt für den weiteren Behandlungs‑, Diagnostik- oder Studienpfad bietet und dabei klinische und wissenschaftliche Interessen synergetisch integriert (Abb. 1). Sektorenübergreifend im Kontext des DAZ beschreibt dabei einerseits die Bahnung der Patiententrajektorien, die sowohl im ambulanten als auch stationären Bereich explizit fachdisziplinenübergreifend erfolgt und eine möglichst über Schnittstellen hinweg integrierte Versorgung der Patienten ermöglichen und mitdenken soll, als auch die Erweiterung der Diagnostik im Hinblick auf nichtärztliche und nichtpsychologische Aspekte.

- Partizipativ erarbeitete Entscheidung über die nächsten Behandlungs‑/Diagnostikschritte durch ausführliche psychometrische und ärztlich-psychologische Diagnostik im Sinne eines diagnoseübergreifenden Störungskonzeptes, inklusive labormedizinischer und elektrokardiologischer Untersuchung.
- Bereitstellung einheitlicher und nach wissenschaftlichen Qualitätsstandards erhobener Daten zur wissenschaftlichen (Sekundär‑)Nutzung bei erfolgter Einwilligung der Patienten (Broad Consent).
- Minimierung von Wartezeiten und Vermeidung von Behandlungssackgassen und -pingpong durch Monitoring und Berücksichtigung der Kapazitäten der gesamten verfügbaren klinischen und externen Angebote in der Behandlungsplanung.
- Frühzeitige Verknüpfung mit therapeutischen Angeboten im Rahmen von Studien und Patientenberatung bezüglich weiterer Studienangebote.

### Eine einheitliche Anlaufstelle

Ein zentrales, klinikweites Kommunikationsangebot bietet die erste Anlaufstelle für Patienten. Während derzeit eine Kontaktaufnahme noch hauptsächlich telefonisch geschieht, werden im Rahmen des Krankenhauszukunftsgesetzes (KHZG) und der Telematikinfrastruktur andere Kommunikations(platt)formen, wie beispielsweise das digitale Patientenportal, an Bedeutung gewinnen und eine direktere Kommunikation und damit Austausch mit Patienten und Einweisern ermöglichen.

Die administrative Aufnahme vor Ort erfolgt zentral und umfasst die Fallanlage und Einholung der Behandlungsverträge. Bereits zu Beginn erfolgt, klar von der administrativen Aufnahme getrennt, die Aufklärung und Einholung des Einverständnisses zum MII Broad Consent durch entsprechend geschultes Personal.

### Psychometrische, apparative und physiologische Diagnostik

Ziel der Diagnostik im DAZ ist eine multimodale, diagnoseübergreifende Charakterisierung der Patienten für klinische und wissenschaftliche Zwecke gleichermaßen. Zu diesem Zweck wurde initial mit Klinikern und Forschern ein Kerndatensatz abgestimmt (Tab. 1), der sich an den Research Domain Criteria (RDoC) orientiert und in breiter Anwendung eine psychologisch-physiologisch-psychiatrische Phänotypisierung entlang des gesamten Spektrums psychischer Gesundheit ermöglicht [2]. Im Rahmen der internen Weiterentwicklung und externen Abstimmung mit den Bemühungen des DZPG unterliegt dieser Kerndatensatz aktuell einer dynamischen Entwicklung. Die Erfassung dieser Daten im Rahmen einer zentralen Einheit ermöglicht hierbei eine hohe Flexibilität bei gleichzeitig hohem Qualitätsstandard. Die Diagnostik erfolgt dabei in einem dreistufigen Verfahren, aktuell aufgeteilt in zwei zeitlich getrennte Termine:

**Table 1:** 

Modul	Art
Basismodul	Soziodemographie
	Psychiatrische Anamnese
	Somatische Anamnese
	Substanzanamnese
	Medikamentenanamnese
	Familienanamnese
	Psychopathologischer Befund (AMDP konform)
Selbst- und Fremdbeurteilungsskalen	Brief Symptom Inventory (BSI-53)
	WHO Disability Assessment Schedule 2.0 (WHODAS)
	Childhood Trauma Screener (CTS)
	Behavioral Avoidance/Inhibition Scale (BIS/BAS-Fragebogen)
	Baratt Impulsiveness Scale (BIS)
	Positive and Negative Affect Schedule (PANAS)
	Emotion Regulation Questionaire (ERQ)
	WHO Fragebogen zum Wohlbefinden (WHO5)
	Clinical Global Impression (CGI)
Neurobiologische Phänotypisierung	Serum, EDTA-, Zitrat‑, Heparinplasma
	EKG
	*EDTA-Vacuette (Genetik und Epigenetik)*
	Tempus-RNA-Vacuette *Blut (Genexpression)*
	MPrage^a^

*Kursiv*: erst nach Einführung des BC erhoben

^a^Abhängig von der klinischen Indikation

Beim ersten Termin erfolgen zunächst die Aufklärung und Einholung klinischer und wissenschaftlicher Einverständniserklärungen sowie die tabletbasierte Erhebung psychometrischer Selbstbewertungsskalen, wobei geschultes Personal unter fachpsychologischer Supervision jederzeit zur Hilfestellung oder Beratung zur Verfügung steht. Die gesamte Erhebung und Dokumentation findet dabei direkt im Krankenhausinformationssystem (KIS) mittels intern programmierter Lösungen statt, um eine nahtlose Integration in die Arbeitsabläufe der klinisch Tätigen zu ermöglichen. Im persönlichen Gespräch werden bereits vorab eingeholte Informationen, wie z. B. Medikationspläne oder Vorbefunde, gegebenenfalls verifiziert und ergänzt. Im zweiten Schritt des ersten Termins erfolgt die apparative und physiologische Diagnostik nach ärztlicher Indikationsstellung und unter fachärztlicher Supervision, bestehend aus Erhebung von Blutdruck, Größe und Gewicht, EKG und klinischer (und zukünftig wissenschaftlicher) Labordiagnostik.

Beim zweiten Termin erfolgt ein diagnostisches Gespräch durch Ärzte oder Psychologen in fortgeschrittener Weiterbildung, welche mit den Strukturen des Hauses gut bekannt sind, den sog. Track-Checkern. Die Erfassung der dabei erhobenen Informationen wird dabei im Rahmen der klinik- und sektorenübergreifenden strukturierten und standardisierten Anamnese notiert. Zuletzt erfolgt unter Einbezug der erfolgten Diagnostik und der Übersicht über aktuelle Kapazitäten eine Beratung mit anschließender partizipativer Entscheidung über die nächsten Schritte. Neben den internen klinischen Angeboten werden darüber hinaus auch externe klinische Angebote sowie wissenschaftliche Studienangebote berücksichtigt. Hat der Patient ein Interesse an letzteren geäußert, wird unter Berücksichtigung der Einschlusskriterien auch hierüber informiert.

Im Anschluss an die Terminserie erfolgt auf Basis einer Patientenbefragung eine Evaluation der Zufriedenheit mit den Terminen, des Grads an erfolgter Information über die Termine und der Einschätzung bezüglich des Vorteils der neuen durchgeführten Diagnostik für ihre individuelle Diagnostik und Behandlung.

### Aufsuchende Diagnostik

Abweichend von oben beschriebenem Ablauf wurde unter Berücksichtigung der Bedürfnisse besonders schwer kranker Patienten, welche im Rahmen der Notfallversorgung des ZI stationär aufgenommen wurden, eine aufsuchende Diagnostik etabliert, welche dieselbe klinisch-wissenschaftliche Phänotypisierung umfasst. Die ärztliche Diagnostik findet dabei im Rahmen des stationären Aufnahmegespräches durch die jeweiligen Stationsärzte statt, um eine Doppeldiagnostik zu vermeiden. Dies ermöglicht auch bislang unterrepräsentierten Patientengruppen die Teilnahme an Forschungsangeboten und ermöglicht retrospektive Studien in sonst schwierig zu erschließenden Settings wie der Notfallsituation.

## Ergebnisse

Seit dem 01.04.2021 wurde das bisher beschriebene Konzept einer zentralisierten, integrierten translationalen Struktur zur Verbesserung der Versorgung und Forschung am ZI zunächst in der Klinik für Psychiatrie und Psychotherapie etabliert. Ziel waren einerseits die Prüfung der generellen Machbarkeit und Akzeptanz, andererseits die Etablierung einer dynamischen Grundstruktur mit der inhärenten Möglichkeit einer kontinuierlich rekursiven und adaptiven Prozessverbesserung im Sinne eines agilen Projekt- und Prozessmanagements.

### Beschreibung der Stichprobe

Insgesamt wurden in dem Zeitraum vom 01.04.2021 bis 31.12.2022 1021 Patienten im DAZ fachpsychiatrisch untersucht; fast die Hälfte der Patienten hatte dabei eine Diagnose aus der Gruppe der affektiven Störungen (47 %), gefolgt von Psychoseerkrankungen (20 %), substanzbezogenen Störungen (15 %) und neurotischen, Belastungs- und somatoformen Störungen (9 %) gemäß ICD-10. Der Vergleich zwischen Patienten, die den elektiven Aufnahmeprozess durch das DAZ durchliefen, mit Patienten, welche im Rahmen der aufsuchenden Diagnostik erfasst wurden, zeigte signifikante Unterschiede zwischen den Gruppen im Hinblick auf die Diagnoseverteilung mit einer deutlichen Überrepräsentation von Patienten mit Psychose im Setting der aufsuchenden Diagnostik.

**Figure 2:**
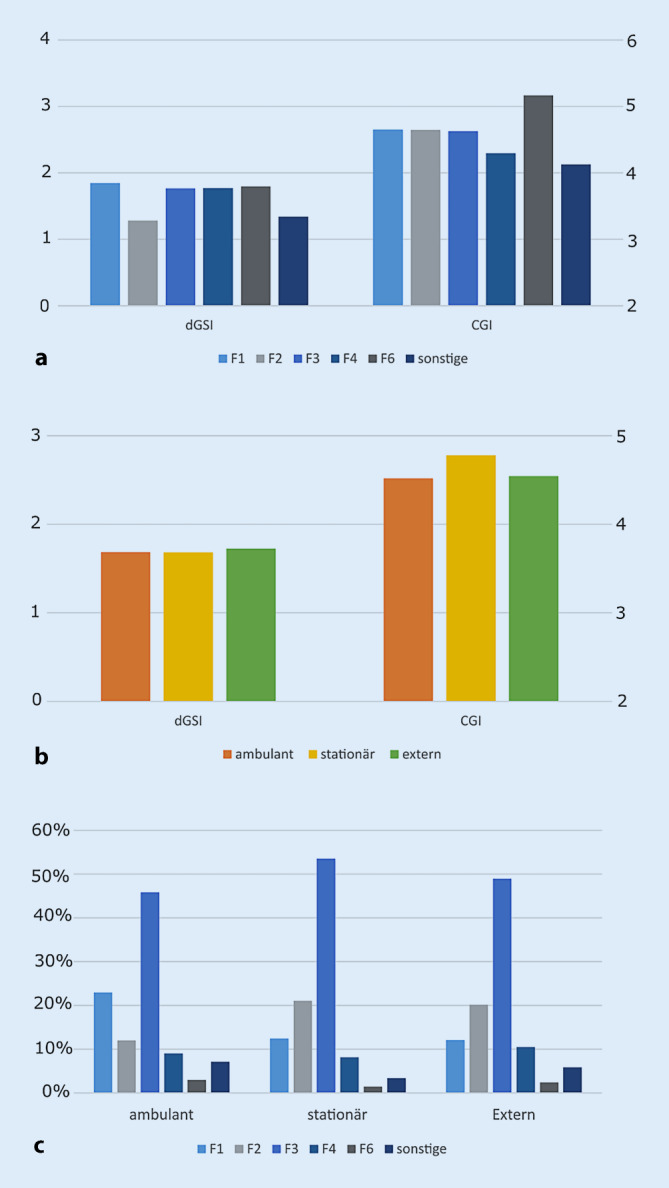

In Tab. 2 ist die Prävalenz der häufigsten Diagnosegruppen im Detail aufgeschlüsselt. Die Geschlechterverteilung war ausgeglichen, mit einem Durchschnittsalter von 38,86 Jahren bei einer Spanne von 18 bis 93 Jahren. Bezüglich der von den Ärzten erhobenen klinischen Krankheitsschwere gemäß der Clinical Global Impression (CGI) wurden die Patienten im Schnitt als mittelgradig bis deutlich schwer erkrankt eingeschätzt (Mittelwert = 4,58), wobei sich in Abhängigkeit von der Diagnosegruppe deutliche Unterschiede (Abb. 2c) zeigten. Interessanterweise zeigte sich nur ein schwacher Zusammenhang zwischen Fremdeinschätzung der Krankheitsschwere mittels CGI und Selbsteinschätzungsinstrumenten wie WHO‑5 oder BSI-53 (CGI-WHO-5: r = −0,268; CGI-BSI-53 GSI: r = 0,113). Eine Subgruppenanalyse in den drei größten Diagnosegruppen zeigte, dass dieser Zusammenhang vor allem von der Gruppe der affektiven Störungen getrieben war, während in der Gruppe der psychotischen Störungen und der Suchterkrankungen kein signifikanter Zusammenhang zwischen Fremd- und Selbsteinschätzungsinstrumenten vorhanden war. Hinsichtlich der Fremdeinschätzung der Krankheitsschwere gemessen am CGI (*p* = 0,128), dem Alter (*p* = 0,309) und dem Geschlecht (*p* = 0,557) zeigten sich keine signifikanten Unterschiede zwischen ambulanter und aufsuchender Diagnostik.

**Table 2:** 

		Alle		Ambulant		Aufsuchend		Vergleich
*N* Gesamt:		1021	100 %	971	95 %	50	5 %	–
Alter	MW	38,86	–	38,74	–	41,08	–	*p* = 0,309
	Spanne	18–93	–	18–93	–	18–84	–	–
	SD	15,842	–	15,750	–	17,552	–	–
Geschlecht:	W	496	49 %	467	48 %	29	58 %	*p* = 0,557
	M	519	51 %	498	52 %	21	42 %	–
Folgebehandlung	Ambulant	271	27 %	266	27 %	5	–	–
	Stationär	217	21 %	209	22 %	8	–	–
	Extern	533	52 %	496	51 %	37	–	–
CGI	N	650	64 %	607	–	43	–	–
	MW	4,58	–	4,59	–	4,36	–	*p* = 0,177
	Spanne	2–7	–	2–7	–	3–6	–	–
	SD	0,77	–	0,77	–	0,79	–	–
WHO 5	N	678	66 %	648	–	30	–	–
	MW	5,54	–	5,40	–	8,47	–	*p* = 0,001
	Spanne	0–25	–	0–25	–	0–25	–	–
	SD	5,18	–	5,03	–	7,33	–	–
BSI-53: GSI	N	693	68 %	663	–	30	–	–
	MW	1,67	–	1,69	–	1,22	–	*p* = 0,001
	Spanne	0–3,77	–	0–3,77	–	0–3,47	–	–
	SD	0,77	–	0,76	–	0,91	–	–
Diagnosen	N	1013	99 %	963	99 %	50	100 %	–
	F0X	25	2 %	24	2 %	1	2 %	–
	F1X	153	15 %	147	15 %	6	12 %	–
	F2X	198	20 %	176	18 %	22	44 %	–
	F31X	32	3 %	30	3 %	2	4 %	–
	F32X	174	17 %	167	17 %	7	14 %	–
	F33X	279	28 %	272	28 %	7	14 %	–
	F4X	95	9 %	93	10 %	2	4 %	–
	F6X	24	2 %	23	2 %	1	2 %	–
	Sonstige	33	3 %	31	3 %	2	4 %	–
	Fehlend	10	1 %	10	1 %	0	0 %	–

*N* Anzahl, *MW* Mittelwert, *SD* Standardabweichung, *W* weiblich, *M* männlich

Eine Subgruppenanalyse ergab, dass sich die Patienten je nach erfolgter Weiterbehandlung in erwartbarer Weise unterschieden (Abb. 2b, c): Patienten, die im Anschluss eine stationäre Behandlung am ZI aufnahmen, waren im Schnitt älter (stationär: 41,3 Jahre, ambulant: 37,9 Jahre, niedergelassene Behandlung: 38,1 Jahre; *p* = 0,03), zeigten ein ausgewogeneres Geschlechterverhältnis (stationär: 50 % weiblich, ambulant: 37 % weiblich, ambulante externe Behandlung: 53 % weiblich; *p* = 0,002) und wurden im Schnitt als schwerer erkrankt eingeschätzt (stationär: 4,8; ambulant: 4,52; externe Behandlung: 4,54; *p* = 0,032) als intern ambulant weiterbehandelte oder nach ambulant extern empfohlene Patienten.

### Akzeptanz des neu etablierten Prozesses

Zur Evaluation des Vorgehens wurde im Sinne einer aktiven Partizipation von Betroffenen eine kontinuierliche Befragung der Patienten durchgeführt. Insgesamt beantworteten 133 Patienten (13 % aller DAZ-Patienten) den Feedbackbogen. Der Großteil dieser (82 %) empfand die durchgeführte ausführliche Diagnostik als (sehr) nützlich für ihren Behandlungsverlauf und war mit dem Ablauf der Termine (sehr) zufrieden (90 %). Verbesserungspotenzial wurde bei vorab erfolgter Information über den Ablauf und Zweck der Termine gesehen, bei dem 15 % der Patienten sich einen ausführlicheren „Fahrplan“ für die Terminserie wünschten.

## Diskussion

Insgesamt zeigte sich, dass die hier etablierte zentrale Aufnahmestruktur mit breiter multimodaler Diagnostik von Patienten eine gute Akzeptanz erfuhr und als zusätzliche Hilfe erlebt wurde und gleichzeitig eine flexible und skalierbare Plattform zur breiten Erfassung klinisch relevanter Forschungsdaten ermöglicht sowie bestehende klinische Infrastrukturen komplementär ergänzt. Unsere Patienten reflektieren dabei eine charakteristische Patientenklientel aus der nichtniedergelassenen Regel- und Pflichtversorgung, sodass von dieser Seite eine breite Anwendbarkeit des Konzepts in der (universitären) Psychiatrie, Psychotherapie und Psychosomatik erwartet werden kann.

Vergleiche mit ähnlichen Ansätzen, ökologisch valide und repräsentative Daten während der Routinebehandlung zu sammeln, wie z. B. durch die Munich Mental Health Biobank (MMHB) oder eine Pilotstudie des Universitätsklinikums Heidelberg, verdeutlichen die unterschiedlichen Vorzüge der verschiedenen Herangehensweisen:

Die z. B. im MMHB-Model gewählte Herangehensweise, welche ähnlich einer Studienrekrutierung auf die Behandlung begleitende bzw. aufsuchende Verfahren setzt, resultierte in einer weniger repräsentativen Stichprobe. Gleichzeitig ermöglicht sie aber eine ausführlichere Erhebung auch behandlungsferner Inhalte bei großer Compliance [7], da es jenseits des klassischen Behandlungskontextes erfolgt. Der von uns gewählte Ansatz bedeutet hingegen eine Umstellung der Behandlungs- und Diagnostikpfade, um eine direkte Integration in klinische Routinen zu ermöglichen und die so gewonnenen Informationen für Patienten und Behandler direkt nutzbar zu machen. Ein Nachteil dieses Ansatzes besteht in der notwendigen Beschränkung auf weniger und wesentlichere Inhalte, da die Rahmenbedingungen durch die eingeschränkteren zeitlichen Kapazitäten in der Routinebehandlung und den zeitlichen, körperlichen und kognitiven Ressourcen der Patienten limitiert sind.

Allen Konzepten gemeinsam ist die technische und prozessuale Herausforderung, die nahtlose Erfassung und Integration der Daten in die existierenden KIS einzubinden. Aktuelle KIS bieten größtenteils noch nicht die in anderen Kontexten gewohnte Benutzerfreundlichkeit, Flexibilität und den Funktionsumfang, um eine auch longitudinale Datenerfassung innerhalb einer Systemumgebung zu ermöglichen. Zwar bieten zahlreiche kommerzielle und nichtkommerzielle Anbieter moderne Electronic-Data-Capture-Systeme mit teils beeindruckendem Funktionsumfang an, deren wechselseitige Integration in die jeweilige KIS-Umgebung kann derzeit allerdings nicht realisiert werden [3, 6]. Erfreulicherweise wurde mit dem KHZG ein Impuls gesetzt, den Funktionsumfang der KIS deutlich zu erweitern, um eine integrierte und benutzerfreundliche Datenerfassung für Patienten und weitere Beteiligte künftig zu ermöglichen. Diese Entwicklung schafft gleichzeitig die Möglichkeit eines aktiveren und direkteren Betroffenenengagements in Forschung und Klinikweiterentwicklung [11].

Die Befunde zur variierenden Korrespondenz von Fremd- und Selbsteinschätzung in unterschiedlichen Diagnosegruppen decken sich einerseits mit der klinischen Beobachtung als auch der Studienlage bezüglich Unterschieden in der Krankheitseinsicht verschiedener psychiatrischer Krankheiten [1, 9]. Insgesamt unterstreichen diese die Wichtigkeit beider Referenzrahmen in der Charakterisierung psychiatrischer Patienten.

### Nächste Schritte

Die positiven Erfahrungen und Rückmeldungen führten zu einer festen Etablierung der bisher entstandenen Struktur im klinischen Routinebetrieb. Aktuell wurde in Kooperation mit der Klinik für Abhängiges Verhalten ein integrierter Prozessablauf entwickelt, der die speziellen Bedürfnisse der Patienten und Behandler dieser Klinik berücksichtigt. Die anvisierte Erweiterung der Struktur um alle Kliniken und Institute des ZI bedingt dabei eine breite und möglichst inklusive Beteiligung aller Patientengruppen mit entsprechend repräsentativen und dem Versorgungsalltag nahen Daten. Hierbei steht auch die Gewinnung einer umfassenderen Feedbackgrundlage im Vordergrund, um die weitere fortlaufende Evaluierung und bedarfsgerechte Anpassung der Struktur gewährleisten zu können.

Parallel erfolgt die Etablierung des Broad Consent mit den dazu notwendigen Strukturen wie der Treuhandstelle und einem Datenintegrationszentrum. Die am Standort ZI bewilligte Form des Broad Consent (Version 1.6d der MII) umfasst dabei unter anderem auch die Möglichkeit zur Entnahme geringer Mengen von Blut für (epi-)genetische Untersuchungen im Forschungskontext. Weiterhin erfolgt derzeit eine dynamische Weiterentwicklung weiterer, auch studienbezogener Phänotypsierungsmodule in enger Abstimmung mit den DZPG- und MII-Partnern. Dabei steht neben der Sammlung weiterer neurobiologischer Modalitäten auch die Integration digitaler und sensorbasierter Systeme im klinischen Alltag im Vordergrund.

Zusammenfassend entsteht mit dem DAZ am ZI eine integrierte translationale Struktur, die aufgrund ihrer engen Einbettung in klinische Prozesse alltagsnahe Daten für Forschung und Krankenversorgung in hoher Qualität erhebt. Die durchgeführte Diagnostik wird von Patienten als hilfreich für ihren Behandlungsverlauf gesehen und ermöglicht eine dynamische Anpassung an Behandlungs- und Diagnostikkapazitäten des ZI. Das DAZ verknüpft im hohen Maße Interessen und Bedürfnisse der Patienten mit der Erhebung wissenschaftlich relevanter Daten, sodass die Teilnahme daran für Patienten möglichst niederschwellig und passiv erfolgen kann.

## Fazit für die Praxis

- Erste Erfahrungen mit dem DAZ liefern Hinweise dafür, dass eine zentralisierte, integrierte translationale Struktur bei guter Patientenakzeptanz zur Verbesserung der Versorgung und Forschung beitragen kann.
- Die aktive Einbeziehung und ein auf die Bedürfnisse der unterschiedlichen Stakeholder (Patienten, Therapeuten und Wissenschaftler) abgestimmtes und interaktives Vorgehen spielen eine wesentliche Rolle.
- Digitale Transformation unterstützt dabei an vielen Schnittstellen durch eine direktere Kommunikation und die Zurverfügungstellung zielgerichteter Informationen translationales Handeln und Denken.
